# Work ability and return-to-work in cancer patients

**DOI:** 10.1038/sj.bjc.6604302

**Published:** 2008-03-18

**Authors:** A G E M de Boer, J H A M Verbeek, E R Spelten, A L J Uitterhoeve, A C Ansink, T M de Reijke, M Kammeijer, M A G Sprangers, F J H van Dijk

**Affiliations:** 1 Coronel Institute for Occupational Heath, Academic Medical Center, University of Amsterdam, Amsterdam, The Netherlands; 2 Cochrane Collaboration Occupational Health Field, Kuopio, Finland; 3 NPVO, Amsterdam, The Netherlands; 4 Department of Radiotherapy, Academic Medical Center, University of Amsterdam, Amsterdam, The Netherlands; 5 Department of Gynaecology and Obstetrics, Academic Medical Center, University of Amsterdam, Amsterdam, The Netherlands; 6 Department of Urology, Academic Medical Center, University of Amsterdam, Amsterdam, The Netherlands; 7 Jan van Breemen Institute, Amsterdam, The Netherlands; 8 Department of Medical Psychology, Academic Medical Center, University of Amsterdam, Amsterdam, The Netherlands

**Keywords:** employment, work ability, return-to-work, longitudinal studies, prospective studies

## Abstract

The extent to which self-assessed work ability collected during treatment can predict return-to-work in cancer patients is unknown. In this prospective study, we consecutively included employed cancer patients who underwent treatment with curative intent at 6 months following the first day of sick leave. Work ability data (scores 0–10), clinical and sociodemographic data were collected at 6 months, while return-to-work was measured at 6, 12 and 18 months. Most of the 195 patients had been diagnosed with breast cancer (26%), cancer of the female genitals (22%) or genitourological cancer (22%). Mean current work ability scores improved significantly over time from 4.6 at 6 months to 6.3 and 6.7 at 12 and 18 months, respectively. Patients with haematological cancers and those who received chemotherapy showed the lowest work ability scores, while patients with cancer of urogenital tract or with gastrointestinal cancer had the highest scores. Work ability at 6 months strongly predicted return-to-work at 18 months, after correction for the influence of age and treatment (hazard ratio=1.37, CI 1.27–1.48). We conclude that self-assessed work ability is an important factor in the return-to-work process of cancer patients independent of age and clinical factors.

Cancer diagnoses in individuals who are still at the working age are becoming more common, with almost half of the adult cancer survivors being younger than 65 years (Short *et al*, 2005). With the sustained improvement in treatment and prognosis of many forms of cancer, an increasing number of survivors of cancer return-to-work following treatment or continue to work during therapy (Hoffman, 2005).

Returning to work is important for both cancer patients themselves and the society. Patients often regard returning to work as a symbol of complete recovery (Spelten *et al*, 2002) and regaining a normal life (Kennedy *et al*, 2007), while from the viewpoint of the society, it is an economic and social imperative to encourage patients to return-to-work whenever possible.

Despite its importance, the impact of cancer and its treatment on work (dis)continuation or resumption has not been studied frequently (Steiner *et al*, 2004). However, a number of studies have documented the impact of cancer on employment and they reported that approximately 60% of the cancer patients return to work within 1–2 years (Spelten *et al*, 2002; Maunsell *et al*, 2004; Bradley *et al*, 2005; Nieuwenhuijsen *et al*, 2006). The return-to-work in cancer survivors seems, therefore, to be problematic in some patients but certainly not in all. Hence, it is important to identify those patients with a higher risk of lasting absence from work and to provide them with the appropriate support and counselling in returning to work.

To examine the factors that would influence this return-to-work process, we previously studied a model based on the assumption that cancer-related symptoms would mediate return-to-work (Spelten *et al*, 2003). However, results showed that diagnosis and treatment were much stronger predictors of return-to-work than cancer-related symptoms such as fatigue, depressive symptoms or cognitive problems. In addition, recent empirical studies have indicated the importance of patients’ expectations of recovery as good predictors of return-to-work and rehabilitation independent of diagnosis and treatment (Ekbladh *et al*, 2004; Verbeek, 2006). Studies in other disorders have also shown that a patient's own assessment of work ability (Reiso *et al*, 2003), expectation of job success (Ekbladh *et al*, 2004) and work recovery expectations (Hogg-Johnson and Cole, 2003; Nieuwenhuijsen *et al*, 2006; Turner *et al*, 2006) do predict return-to-work.

A theory that could explain these mechanisms is the well-known Leventhal's ‘model of illness representations’, which states that people's cognitive representations of illness exert an important influence on their strategies for coping, which in turn influence illness outcomes (Leventhal *et al*, 1984). It has been shown in other diseases such as multiple sclerosis, rheumatoid arthritis and kidney disease (Vaughan *et al*, 2003; Carlisle *et al*, 2005; Fowler and Baas, 2006) that, on the basis of this model, the functional outcome might be worse or better, irrespective of the objective medical seriousness of the illness. This strongly suggests that the ideas a cancer patient has about the disabilities that might result from the diagnosis and treatment will encourage or hinder his or her return-to-work.

With these new insights, our data were reanalysed with the focus on the patients’ assessments of work ability as predictor of return-to-work. In our earlier publication on return-to-work of cancer survivors, we did not use information on the self-assessed ability to work because at the time it was outside the focus of our study (Spelten *et al*, 2003).

The aim of the current study is therefore (1) to examine any change in work ability scores in cancer patients over time and to study differences among patient groups and (2) to assess the extent to which self-assessed work ability predicts return-to-work among cancer survivors independent of diagnosis, treatment and cancer-related symptoms.

## MATERIALS AND METHODS

### Patients

Eligible patients had to be between 18 and 58 years to have a primary diagnosis of cancer, to be in paid employment at the time of diagnosis, to be within 4–6 months following their first day of sick leave, and to have had treatment with curative intent. They were consecutively recruited in three hospitals in The Netherlands where the attending physician obtained the patients’ informed consent. The study has been carried out with the approval of the hospitals’ medical ethical committees.

Questionnaires were distributed three times to the patients, at entry into the cohort and 6 and 12 months later, to obtain information on their return-to-work, diagnosis, treatment, work ability and cancer-related symptoms. Details of the design and material of this prospective cohort study have been reported earlier (Spelten *et al*, 2003). The data were collected between 1998 and 2002. For the current study, data on return-to-work and work ability were collected at study entry and 6 and 12 months later. Data on work load, work stress, cancer-related factors and sociodemographic factors had been collected at baseline. All questionnaires were mailed to the patients’ homes.

### Measures

#### Return-to-work

Data on return-to-work were measured on the basis of two measures: time to return-to-work after sick leave and rate of return-to-work at a specific point in time. All patients in The Netherlands typically have access to sick leave. Time to return-to-work at 18 months after the first day of sick leave was calculated as the number of days between the first date of sick leave and the first day the patient returned to work. Any kind of work resumption qualified as a return-to-work, irrespective of the number of hours that the patients worked prior to their diagnosis. In addition, patients were asked to indicate if they were still on sick leave (yes/no) at 6, 12 and 18 months following their first day of sick leave.

#### Work ability, work load and work stress

Current work ability was measured with the first three items from the Work Ability Index (WAI) (Ilmarinen and Tuomi, 1993, p 142; Tuomi *et al*, 1998), which is a reliable and valid measure of work ability (Ilmarinen and Tuomi, 1993, p 142; de Zwart *et al*, 2002). First, current work ability was assessed by asking the patients to estimate their current work ability compared with their lifetime best (0=cannot work at all to 10=best ever). In addition, we asked the cancer patients to rate both their current physical and mental work ability in relation to job demands (0=very low to 5=very high).

Physical workload was measured with a seven-item scale and work stress with an 11-item scale from the Dutch Questionnaire on Experience and Judgement of Work (VBBA) (van Veldhoven *et al*, 2002). Patients were asked to assess their levels of workload and work stress for the work situation prior to diagnosis. The scores range from 0 to 100, with higher scores indicating a higher level of physical work and more work stress, respectively.

#### Cancer-related and sociodemographic factors

Information about diagnosis and treatment was reported by the patients. Twenty-two different diagnoses were then grouped according to cancer site into (1) breast cancer, (2) haematological oncology, (3) gastrointestinal cancer, (4) cancer of the female genitals, (5) genitourological cancer and (6) other types of cancer. Treatments were classified into three categories: (1) surgery, (2) radiotherapy or radiotherapy plus surgery and (3) chemotherapy or chemotherapy plus radiotherapy and/or surgery.

We measured cancer-related complaints with validated questionnaires and converted all scores to a scale ranging from 0 to 100, with higher scores indicating more complaints (Spelten *et al*, 2003). The following complaints were measured: physical cancer-related complaints (de Haes *et al*, 1990), general fatigue (Smets *et al*, 1995), sleep quality (Buysse *et al*, 1989), depressive symptoms (Radloff, 1977), psychological distress (de Haes *et al*, 1990), cognitive dysfunction (Broadbent *et al*, 1982) and global quality of life (de Haes *et al*, 1990).

Further information was enquired concerning marital status (single, married, cohabitating or other), having children in the household, age, gender, education (lower education, high school, college/university) and work hours per week before the diagnosis of cancer.

## STATISTICAL ANALYSIS

The work ability scores measured at 6, 12 and 18 months after the first day of sick leave were analysed with the mixed-model procedure based on repeated measurements to examine any change in work ability scores over time. We also used the mixed-model procedure to analyse any differences over time in work ability scores between several patient groups: age groups (18–27, 28–37, 38–47 and 48–58 years), education groups, men and women, diagnosis groups and treatment groups. Time, group and time*group interaction effects were considered fixed effects and an autoregressive covariance structure was selected because of correlated work ability scores over time. In case of a statistically significant main effect, *post hoc* analyses were performed between time points and between groups with pairwise comparisons based on the use of the mean difference of the estimated marginal means.

To examine whether self-assessed work ability can predict return-to-work in cancer patients a year later, taking the impact of clinical-, work- and subject-related factors into account, we used a two-step procedure. First, univariate analyses using Kaplan–Meier analyses were performed for the relationship between time taken to return-to-work (in days) at 18 months and each of the predictive factors measured at baseline (on average 6 months after the first day of sick leave): current work ability, mental work ability, physical work ability, physical work load, work stress, physical complaints, fatigue, sleep impairments, depression, psychological distress, cognitive dysfunction, age, gender, education and the clinical factors (diagnosis and treatment type). Next, we analysed the impact of work ability in addition to personal and clinical factors in a multivariate Cox regression analysis. We entered all variables for which the log-rank test returned a *P*-value⩽0.10 into a Cox regression analysis with forward selection of variables. With this method, the best predictors of future return-to-work are selected (Altman, 1991). Because it was possible for patients to return to work before our first measurement at 6 months, we repeated both analyses with the exclusion of patients who had returned before 6 months. Since this is a survival analysis, hazard ratios (HRs) usually indicate the risk of dying, while in our case the event is returning to work. Therefore, an HR higher than one indicates the higher ‘risk’ of return-to-work.

Alpha was set at 0.05 unless stated otherwise and all tests were two-sided. Analyses were conducted with SPSS 13.

## RESULTS

The first questionnaire was completed by 235 of the 264 eligible patients (a response of 89%), while a total of 29 patients declined participation in this study. The second questionnaire at 6 months follow-up was completed by 221 of the 235 participating patients (a follow-up response of 94%). At 12 months of follow-up, the questionnaire was returned by 195 patients (an 83% follow-up response and 74% of the initially eligible patients), while 25 patients refused to return the questionnaire, 13 patients had died and 2 questionnaires got lost in the mail.

Table 1 shows sociodemographic and cancer-related characteristics at 6 months after the first day of sick leave. Half of the patients had either breast cancer (26%) or cancer of the female genitals (22%), while another 22% of the patients had been diagnosed with genitourological cancer. Before diagnosis and treatment, patients worked an average of 34 h per week, and 6 months after the diagnosis, 46 patients (24%) had already returned to work or had continued working. Data on work hours per week, children, fatigue, depression, sleep problems, physical complaints, cognitive dysfunction, psychological distress, work load and work stress have been reported previously (Spelten *et al*, 2003).

Table 2 depicts the mean values of current work ability at 6, 12 and 18 months after the first day of sick leave; the values improved significantly over time (*P*<0.001) from 4.6 at 6 months to 6.3 at 12 months and to 6.7 at 18 months. *Post hoc* analyses of work ability scores showed that all three time points were significantly different from each other (*P*<0.001 to *P*=0.035). All age groups improved over time (*P*<0.001) with the 28- to 37-year-old patients increasing most from 4.8 to 7.5. No differences in work ability scores were, however, found between age groups (*P*=0.12). Work ability scores of both men and women improved over time (*P*<0.001), but women improved more (*P*=0.002). Male patients showed higher work ability scores at 6 months (5.8 *vs* 3.8, *P*<0.0001), but not at 12 months (6.8 *vs* 6.0, *P*=0.053) or at 18 months (6.9 *vs* 6.7, *P*=0.52). Higher educated patients seemed to have higher work ability scores, but the differences were not statistically significant (*P*=0.13). With regard to diagnosis, we found significant differences between the different diagnosis groups (*P*<0.001). The haematological oncology patients showed the significantly (*P*<0.001) lowest scores of 3.3, 4.5 and 5.0 at 6, 12 and 18 months, respectively. The patients with genitourological cancer had the highest scores of 6.9 and 7.8 at 6 and 12 months (*P*<0.001), and the patients with gastrointestinal cancer scored the highest work ability of 7.6 at 18 months (*P*<0.001). Patients with cancer of the female genitals and breast cancer patients improved most over time (*P*=0.01).

**Figure 1:**
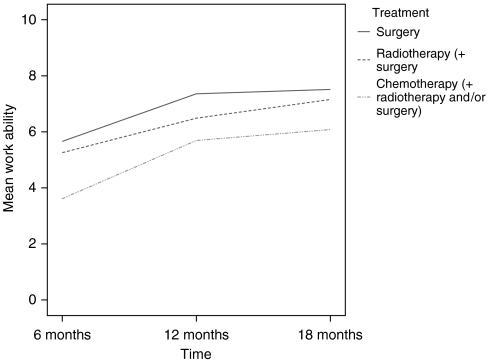
Mean value of current work ability at 6, 12 and 18 months after the first day of sick leave for the three treatment combinations: surgery; chemotherapy or chemotherapy plus radiotherapy and/or surgery; and radiotherapy or radiotherapy plus surgery (*n*=195).

Figure 1 shows the work ability scores for the three treatment combinations: (1) surgery; (2) chemotherapy or chemotherapy plus radiotherapy and/or surgery; and (3) radiotherapy or radiotherapy plus surgery, over time. Analyses revealed that scores improved over time for all three groups and that the group of patients that received chemotherapy or chemotherapy plus radiotherapy and/or surgery consistently showed lower work ability scores than the group that received surgery or radiotherapy (plus surgery) (*P*<0.001). Improvement was not statistically different in the three groups (*P*=0.45).

At 6 months after diagnosis, 24% of patients had returned to work, at 12 months 50%, and at 18 months 64% had returned. Results of univariate analyses using the Kaplan–Meier analyses showed that the time taken to return-to-work measured at 18 months was related to the following factors measured at 6 months: current work ability, mental work ability, physical work ability, quality of life, fatigue, physical complaints, cognitive functioning, age, physical work load, work stress, gender, diagnosis and treatment (at the *P*⩽0.10 level). Sleep impairments, depression, psychological distress and education did not significantly predict return-to-work. Results of the analysis without the 46 patients who had already returned to work at 6 months showed the same factors except for gender, which did not significantly predict return-to-work.

**Figure 2:**
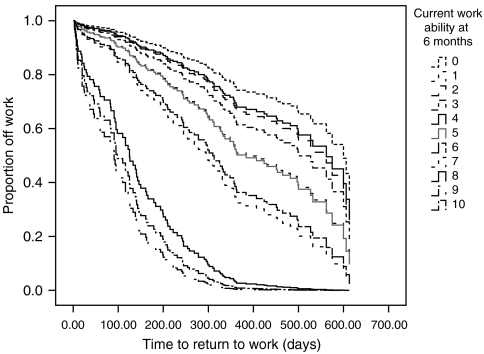
Plot of the work ability scores measured at 6 months in relation to time to return-to-work, after adjustment for age and treatment (*n*=195).

The factors that were predictive for return-to-work at 18 months were entered in the Cox regression with a forward selection to identify the strongest predictors of return-to-work. Results in Table 3 show that in the final model, age, current work ability and treatment are still significant. Current work ability, physical work ability and mental work ability were highly correlated and, therefore, only current work ability remained in the model. Likewise, treatment and diagnosis were highly correlated, and only treatment was selected for the final model. Patients treated with surgery alone had the highest chance of returning to work quickly. Those who were treated with radiotherapy or radiotherapy plus surgery had an HR of 0.63 (95% CI: 0.39–1.0), corrected for age and work ability, of returning to work and were thus 1.6 times more likely to stay off work than patients with surgery alone. Patients treated with chemotherapy, either alone or in combination with other treatment modalities, had an HR of 0.41 (95% CI: 0.25–0.69) and their risk of staying off work was therefore 2.4 times higher than patients treated with surgery alone, corrected for age and work ability. For current work ability itself, every 1 point increase on the 11-point scale meant a 1.37 higher chance of returning to work earlier, after correction for the influence of age and treatment. Figure 2 shows the plot of the work ability scores in relation to return-to-work after adjustment for age and treatment. The plot shows that of the patients with the lowest work ability scores (0–5) at 6 months after the first day of sick leave, the majority (55–80%) did not return to work in the first year after diagnosis. Patients with very high work ability scores (8, 9, 10) did usually return to work within half a year, while virtually all of these high-scoring patients were back at work after the first year.

Results of the analysis without the 46 patients who had already returned to work at 6 months showed that in this model the strongest predictors of return-to-work were not only work ability (HR=1.23; CI, 1.12–1.36), treatment (chemotherapy HR=0.33; CI, 0.18–0.60; radiotherapy HR=0.52; CI, 0.29–0.95) and age (HR=0.67; CI, 0.53–0.86) but also mental work ability (HR=1.41; CI, 1.05–1.89) and cognitive dysfunction (HR=1.03; CI, 1.01–1.05).

## DISCUSSION

The aim of our study was to examine changes in work ability scores in cancer patients over time and to study differences between patient groups and, furthermore, to assess the extent to which self-assessed work ability predicts return-to-work among cancer survivors independent of diagnosis, treatment and cancer-related symptoms.

We found that the cancer patients’ work ability scores at 6, 12 and 18 months after the first day of sick leave improved significantly over time. Men scored higher on work ability than women but no differences were found between age or education groups. Furthermore, the haematological oncology patients and the patients who received chemotherapy or chemotherapy plus radiotherapy and/or surgery consistently showed lower work ability scores. Finally, self-assessed work ability 6 months after the first day of sick leave proved to be a strong predictor of later return-to-work in cancer survivors independent of age and therapy.

This is the first longitudinal study in which the impact of work ability on return-to-work has been established in a systematic way. The cohort has been followed for a considerable amount of time, the number of patients lost to follow up was relatively small and all factors have been measured with validated instruments.

In our study, the mean current work ability scores at 6, 12 and 18 months after the first day of sick leave were 4.6, 6.3 and 6.7, respectively. Although we found a significant improvement of current work ability, these scores are lower than the average current work ability score of 7.9 found by Pohjonen (2001) in a sample of female home care workers in the age group 40–44 years old with an average of two diagnosed benign diseases. It might be possible that work ability scores in cancer patients will improve still further 2 years after the diagnosis or that their work ability scores might deteriorate because cancer has a larger impact on work ability than other diseases.

Research on the effect of cancer diagnosis and treatment on work ability is scarce; however, studies have shown recently that most patients are employed but that both physical and mental work ability can deteriorate owing to cancer (Gudbergsson *et al*, 2006; Steinbach *et al*, 2006; Kennedy *et al*, 2007; Taskila *et al*, 2007). Patients in the recent study of Kennedy *et al* (2007), who were 1–10 years after diagnosis, reported that they had difficulties in coping and concentrating, and they worried about their reduced capability. In the comparative study of Gudbergsson *et al* (2006), it was found that cancer patients 2–6 years after diagnosis, who had returned to work after curative treatment, reported significantly poorer physical and mental work capacity compared to employed matched controls from the general population. Most survivors of glioblastoma in the study of Steinbach *et al* (2006) also thought that their work ability was impaired. According to Taskila *et al* (2007), 26% of cancer survivors reported that their physical work ability had deteriorated and 19% that their mental work ability had deteriorated owing to cancer diagnosis and treatment. However, the work ability as measured with the WAI of these cancer survivors did not differ from that of a group of healthy referent persons. This is probably also caused by the fact that all their survivors with breast cancer, lymphoma and prostate cancer had already returned to work and that they were long-term survivors who had been diagnosed with cancer 2–6 years before the time of the questionnaire. This could also explain the differences in mean work ability scores between their study and the patients in our study. For men in their study, the work ability scores were 8.0 (for prostate cancer) to 8.9 (for testicular cancer) compared to 6.9 for the men in our study 18 months after the first day of sick leave. Our female patients scored 6.7 at the end of follow-up compared to 8.2 (for breast cancer) and 8.5 (for lymphoma) in the Finnish study by Taskila *et al* (2007). Our study also showed that men initially showed higher scores of work ability, while women improved faster and no differences were found after 1 year. It might be possible that women, who were mainly diagnosed with breast cancer, received more chemotherapy, which would have prolonged the treatment period. Another explanation might be that women could have more household activities than men and that they take these into account when judging their work ability.

Our study indicated work ability as an independent predictor for return-to-work, while quality of life was only found to be predictive of time until return-to-work in the univariate analyses. The same result was found earlier for Norwegian patients with back disorders who had been certified as sick (Reiso *et al*, 2003). The authors of that study suggested that work ability questions may be related more to function in a setting of sickness certification than a global quality of life question and therefore be more predictive.

In an earlier analysis (Spelten *et al*, 2003), we found that fatigue at 6 months predicted a longer sick leave with an HR of 0.71, adjusted for diagnosis, treatment, age and gender. Our present study indicated that fatigue was only a predictive factor of return-to-work in the univariate analyses but not in the multivariate analyses, which included work ability. Because work ability and fatigue were correlated, only work ability remained in the model as the better predictor of return-to-work. Other studies have also found that fatigue influenced conditions of employment and productiveness (Hofman *et al*, 2007). Results of the univariate analyses without the 46 patients who had already returned to work at 6 months showed that gender was not a significant factor anymore. This might be caused by the fact that most of these returned patients were men (65%) and were diagnosed with testes or prostate cancer (48%). In the model of best predictors of return-to-work without those patients who had returned early, the factors mental work ability and cognitive dysfunction were included. This could imply that for patients who do not return early, the mental and psychological factors become more dominant in relation to return-to-work.

Leventhal's ‘model of illness representations’ states that people's cognitive representations of illness play an important role in influencing their strategies for coping, which in turn influence illness outcomes (Leventhal *et al*, 1984). On the basis of this model, the functional outcome might be worse or better, irrespective of the objective medical seriousness of the illness. Our results are congruent with this model. Irrespective of age, diagnosis, treatment, quality of life, fatigue, and physical or psychological complaints, self-assessed work ability strongly predicted future return-to-work. This indicates that the ideas a cancer patient has about his or her work disabilities that result from the diagnosis and treatment of cancer are a reflection of the true work capabilities. Therefore, the self-reported work ability could be important in encouraging or hindering his or her return-to-work.

Employment outcomes can be improved with innovations in treatment and with clinical and supportive services aimed at better management of symptoms, rehabilitation and accommodation of disabilities (Steiner *et al*, 2004). A recent study of Bouknight *et al* (2006) showed that a high percentage of employed breast cancer patients returned to work after treatment and that workplace accommodations played an important role in their return. Therefore, interventions should be developed to enable cancer survivors to return to work or to succeed in other appropriate employment, because no such interventions aimed at work do exist at the present. These interventions should aim especially at patients who indicate that their work ability is diminished, at older patients and at those treated with chemotherapy, since they are at the greatest risk of prolonged work absence. Clinicians could play an important role in detecting those patients at risk because our study has shown that the indication of patients with possible return-to-work problems can be assessed very early in the treatment process when they have diminished self-reported work ability. Physicians could help patients in the return-to-work process and therefore help in improving their quality of life by asking patients if they have returned to work or are experiencing problems in the return-to-work process. If so, referral to occupational specialists could be considered.

In conclusion, the work ability of cancer patients who work at the time of their diagnosis is severely impaired in the first months after the first day of sick leave, but it does improve significantly in the months afterwards. Self-assessed work ability 6 months after the first day of sick leave proved to be a strong predictor of later return-to-work in cancer survivors independent of age and therapy.

## Figures and Tables

**Table 1:** Sociodemographic and cancer-related characteristics at baseline, on average 6 months after the first day of sick leave

***N*=195 patients**	***n* (%)**
Age (mean (s.d.))	42.2 (9.3)
Sex (male)	78 (40%)
Returned-to-work	46 (24%)
	
*Education*	
Lower	52 (27%)
High school	83 (42%)
College/university	60 (31%)
	
*Marital status*	
Single	24 (12%)
Married/cohabiting	160 (82%)
Divorced	8 (4%)
Widower	3 (2%)
	
*Diagnosis*	
Breast cancer	51 (26%)
Haematological oncology	24 (12%)
Gastrointestinal cancer	23 (12%)
Cancer of the female genitals	43 (22%)
Genitourological cancer	43 (22%)
Other	11 (6%)
	
*Treatment*	
Surgery	41 (21%)
Chemotherapy or chemotherapy plus radiotherapy and/or surgery	88 (45%)
Radiotherapy or radiotherapy plus surgery	66 (34%)

**Table 2:** Mean value of current work ability according to sociodemographic and disease-related factors at 6, 12 and 18 months after the first day of sick leave

	**Workability score^a^ (mean (s.d.))**		
***N*=195 patients**	**6 months**	**12 months**	**18 months**
All patients^b^	4.59 (3.2)	6.31 (2.7)	6.74 (2.7)
			
*Age*			
18–27 years	5.33 (3.6)	5.71 (3.9)	7.27 (2.9)
28–37 years	4.81 (3.4)	6.80 (2.5)	7.51 (2.4)
38–47 years	4.53 (3.2)	6.65 (2.3)	6.80 (2.3)
48–58 years	4.31 (3.0)	5.73 (2.8)	5.97 (3.1)
			
*Sex* c			
Male	5.76 (3.0)	6.78 (2.6)	6.91 (2.8)
Female	3.83 (3.1)	6.00 (2.7)	6.65 (2.6)
			
*Education*			
Lower	3.90 (3.3)	6.14 (3.0)	6.26 (3.2)
High school	4.51 (3.0)	6.31 (2.6)	6.77 (2.5)
College/university	5.33 (3.4)	6.46 (2.6)	7.15 (2.6)
			
*Diagnosis* c			
Breast cancer	3.59 (3.1)	5.90 (2.3)	6.49 (2.5)
Haematological oncology	3.29 (3.0)	4.46 (3.4)	4.95 (3.6)
Gastrointestinal cancer	5.52 (2.7)	6.95 (2.1)	7.57 (1.6)
Cancer of the female genitals	3.91 (3.1)	6.29 (2.9)	7.00 (2.6)
Genitourological cancer	6.86 (2.5)	7.79 (1.7)	7.33 (2.7)
Other	4.09 (3.4)	5.36 (2.9)	6.55 (2.5)

a Range 0–10; 10 indicating best work ability ever.

b Work ability score change over time: *P*<0.01.

c Difference between groups: *P*<0.01.

**Table 3:** Cox regression analysis on return to work

	**Time to return to work**	
***N*=195**	**Hazard ratio**	**95% CI**
Age, 10-year categories	0.78	0.65–0.94
Current work ability	1.37	1.27–1.48
		
*Treatment*		
Surgery (reference)	1.00	
Chemotherapy plus radiotherapy and/or surgery	0.41	0.25–0.69
Radiotherapy/radiotherapy plus surgery	0.63	0.39–1.0

95% CI=95% confidence interval.
